# Challenges in amphetamine medication availability for individuals with ADHD: a narrative review of the current state of evidence

**DOI:** 10.3389/fpsyt.2025.1624590

**Published:** 2025-07-23

**Authors:** Mansour M. Alotaibi, Naif Z. Alrashdi, Bakriah Alzubaidi, Marzouq K. Almutairi, Sultan A. Alanazi, Anwar B. Almutairi, Mohammed M. Alqahtani

**Affiliations:** ^1^ Department of Rehabilitation, Faculty of Applied Medical Sciences, Northern Border University, Arar, Saudi Arabia; ^2^ King Salman Center for Disability Research, Riyadh, Saudi Arabia; ^3^ Department of Physical Therapy and Health Rehabilitation, College of Applied Medical Sciences, Majmaah University, Al-Majmaah, Saudi Arabia; ^4^ Health and Basic Sciences Research Center, Majmaah University, Majmaah, Saudi Arabia; ^5^ Department of Rehabilitation, Ministry of National Guard Health Affairs, Taif, Saudi Arabia; ^6^ Department of Physical Therapy, College of Applied Medical Sciences, Qassim University, Buraydah, Saudi Arabia; ^7^ Department of Physical Therapy, Faculty of Allied Health Sciences, Kuwait University, Jabriya, Kuwait; ^8^ Department of Respiratory Therapy, College of Applied Medical Sciences, King Saud bin Abdulaziz University for Health Sciences, Riyadh, Saudi Arabia; ^9^ King Abdullah International Medical Research Center, Riyadh, Saudi Arabia

**Keywords:** attention deficit hyperactivity disorder, amphetamine, lisdexamfetamine, psychostimulants, access, ADHD

## Abstract

**Background:**

Attention-deficit/hyperactivity disorder (ADHD) is a prevalent neurodevelopmental disorder affecting individuals across various age groups. Access to amphetamine (AMPH) stimulant is a critical component of evidence-based care for individuals with ADHD. In Saudi Arabia, despite clinical guidelines endorsing their use, the availability of AMPH-based stimulants remains limited.

**Objective:**

This narrative review aims to explore the current regulatory and policy environment influencing AMPH medication availability for individuals diagnosed with ADHD in Saudi Arabia.

**Methods:**

A narrative review methodology was adopted following the Scale for the Assessment of Narrative Review Articles guidelines. A literature search was conducted in PubMed, EBSCO, and PsycINFO using defined search terms related to ADHD, psychostimulants, and Saudi Arabia. Additional grey literature from key regulatory bodies, such as the Saudi Food and Drug Authority (SFDA), the Ministry of Health (MOH), and the Saudi ADHD Society, was also reviewed. Thirteen articles and reports met the eligibility criteria and were included for qualitative synthesis.

**Results:**

Methylphenidate is the predominant stimulant prescribed for ADHD, while AMPH-based medications, such as lisdexamfetamine, are under prescribed due to regulatory restrictions, limited formulary inclusion, and supply inconsistencies. Policy reports from national institutions highlight persistent barriers to AMPH access, despite their inclusion in recent clinical practice guidelines. Prescription trends suggest significant treatment gaps for AMPH stimulants.

**Conclusion:**

The available evidence suggests a likely shortfall in AMPH-based medications. in Saudi Arabia, despite global and local evidence supporting their efficacy. Our findings suggest the need for enhancing regulatory pathways regarding AMPH access and availability, which requires policy interventions targeting regulatory reform, formulary expansion, and improved awareness among AMPH prescribers.

## Introduction

Attention-deficit/hyperactivity disorder (ADHD) is a prevalent neurodevelopmental disorder that affects 5-8% of children and adolescents worldwide (1, 2) and 6.8% in adults (3), with substantial variation across regions and diagnostic criteria (4). Approximately, 2.6% of childhood ADHD cases persist into adulthood (3), contributing to the increased prevalence of ADHD among adults (3). In Saudi Arabia, the prevalence of ADHD among adolescents (12–18 years) and adults was estimated to be 11.29% (5), however, no accurate estimates of the national prevalence of ADHD among children (6–12 years) were researched, which warrants future studies. Typically, ADHD can be categorized based on the dominancy of its major symptoms, including inattention (predominantly inattentive) and hyperactivity-impulsivity (predominantly hyperactive/impulsive) or both (combined) (6). These ADHD symptoms were responsible for negative impacts on the individual’s personal life, contributing to academic underachievement (7, 8), impaired professional performance and unemployment (9), and/or suboptimal quality-of-life (10–12).

The first line of pharmacological treatment for children and adults with ADHD is psychostimulant medications which include methylphenidate-derived (MPH) and amphetamine-derived medications (AMPH) (13). Meta-analyses continue to show that AMPH-based stimulants, including mixed amphetamine salts and lisdexamfetamine demonstrate relatively large effect size in reducing ADHD symptoms, with standardized mean difference (SMD) ranging from-0.64 to -0.89 (14, 15). Generally, psychostimulant medications work on increasing dopamine and norepinephrine levels in the brain, thereby enhancing attention and impulse control (16). MPH increases norepinephrine and dopamine levels in the presynaptic axon by blocking the reuptake of these neurotransmitters. However, AMPH further increases secretion of these neurotransmitters through the inhibition of vesicular monoamine transporter 2 as well as through interruption of the electrochemical gradients necessary for vesicular transporter function (17). For individuals who are stimulants intolerant or have contraindications, non-stimulant alternatives such as atomoxetine, guanfacine, and clonidine are treatment options (18).

Despite the benefits of pharmacotherapy, concerns regarding their side effects, long-term efficacy, and potential misuse necessitate careful clinical assessment, and ongoing monitoring (19). Common side effects of psychostimulant medications include appetite suppression, sleep disturbances, and increased heart rate, meanwhile non-stimulant medications may cause fatigue, dizziness, or gastrointestinal discomfort (14). However, it has been shown that if left untreated, individuals with ADHD may face significant functional impairments across multiple domains of life, including academic (20), occupational (21), psychological (22, 23), and social aspects (24). Therefore, an evidence-based approach that includes individualized treatment planning, regular follow-ups, and consideration of non-pharmacological interventions is essential for optimizing outcomes in ADHD management. Such approach should include the provision of effective medications, like AMPH-derived medications, which would help in controlling ADHD symptoms.

Standard ADHD clinical practice guidelines (CPGs), such as the American Academy of Pediatrics (25) and the National Institute for Health and Clinical Excellence (NICE) (26), promote the use of AMPH-based stimulants for children aged 6 years and older. Limited access to AMPH-based stimulants for individuals with ADHD can lead to significant negative health outcomes and impose additional burdens on the healthcare system. Evidence found that using AMPH-based stimulants, in addition to effectively reducing core ADHD symptoms, have been associated with lower risks of suicidal behavior and psychiatric hospitalizations (27, 28).

According to the Centers for Disease Control and Prevention, restricted access to these medications can result in untreated or inadequately managed ADHD, leading to adverse outcomes including social and emotional impairments, increased risk of substance use, unintentional injuries, and suicide (29). From a healthcare system perspective, medication shortages and access barriers can force patients to seek alternative, often unregulated, sources for their medications, increasing the risk of exposure to counterfeit substances and associated health complications (29, 30). Furthermore, the lack of appropriate medication management may result in strain on healthcare resources due to the exacerbation of ADHD-related complications, thus elevating overall healthcare costs (29). Securing availability of AMPH-based stimulants in clinical practice is essential for optimizing health outcomes in individuals with ADHD and reducing the broader impact on the healthcare system.

Regulatory actions play a pivotal role in addressing medication shortages and prevent reliance on unregulated sources. Given the limited access of AMPH-based stimulants in Saudi Arabia, this study aims to synthesize existing knowledge on amphetamine availability for ADHD, explore the regulatory processes and healthcare system challenges that contribute to access barriers, and provide a policy recommendations on improving medication availability for this population.

## Methods

### Research question and study design

We reported this review in accordance with the Scale for the Assessment of Narrative Review Articles guidelines (SANRA) (31). SANRA is a validated tool designed to improve the quality, transparency, and reproducibility of narrative reviews (31). Key SANRA domains addressed in this review include justification of the article’s importance, appropriate referencing, transparent literature search, scientific reasoning, and balanced presentation of relevant literature (31). The aim of this review was to broadly understand the current literature on the availability of AMPH-derived stimulants for individuals with ADHD in Saudi Arabia. We selected to perform a narrative review after scoping searches revealed that the evidence base comprised a small number of observational studies and substantial grey literature (national guidelines, formulary documents, regulatory circulars). Given this heterogeneity, a formal systematic review would have yielded few studies and excluded critical policy sources. Narrative synthesis, guided by the SANRA quality framework (31), therefore offered the most suitable method for integrating multidisciplinary evidence and generating policy-relevant conclusions. In this narrative review, we summarized and reported the available published work as well as grey literature regarding challenges related to AMPH-based stimulants access and availability for physicians when treating individuals diagnosed with ADHD.

### Search strategy

We comprehensively searched the current available literature for published work including PubMed, PsycINFO, and EBSCO from inception until March 1, 2025. Key terms (with their terminologies) included (ADHD OR “attention deficit hyperactivity disorder”) AND (psychostimulants OR amphetamine OR dextroamphetamine) AND (Saudi Arabia). Details on search terms were provided in **Supplementary Table 1**. Additionally, we searched other potentials sources for grey literature work such as regulatory agencies’ websites including the Saudi Ministry of Health (MOH; https://www.moh.gov.sa/Pages/Default.aspx(, Saudi Food and Drug Authority (SFDA; https://www.sfda.gov.sa/en), and the Saudi ADHD Society (https://adhd.org.sa/en/). The reference lists of the included studies were also carefully examined by two reviewers (the first author; MMA and the second author; NZA) manually to locate other potentially relevant studies.

### Eligibility criteria

We included peer-reviewed articles that included participants diagnosed with ADHD. We also included national guidelines, formulary documents, regulatory circulars, and relevant grey literature that addressed AMPH-based treatment in ADHD and included data or commentary specific to Saudi Arabia. Articles without regional specificity, editorials, and non-medication-focused reports were excluded. Articles were included if they were written in English and Arabic.

### Study selection and data extraction

**Figure 1:**
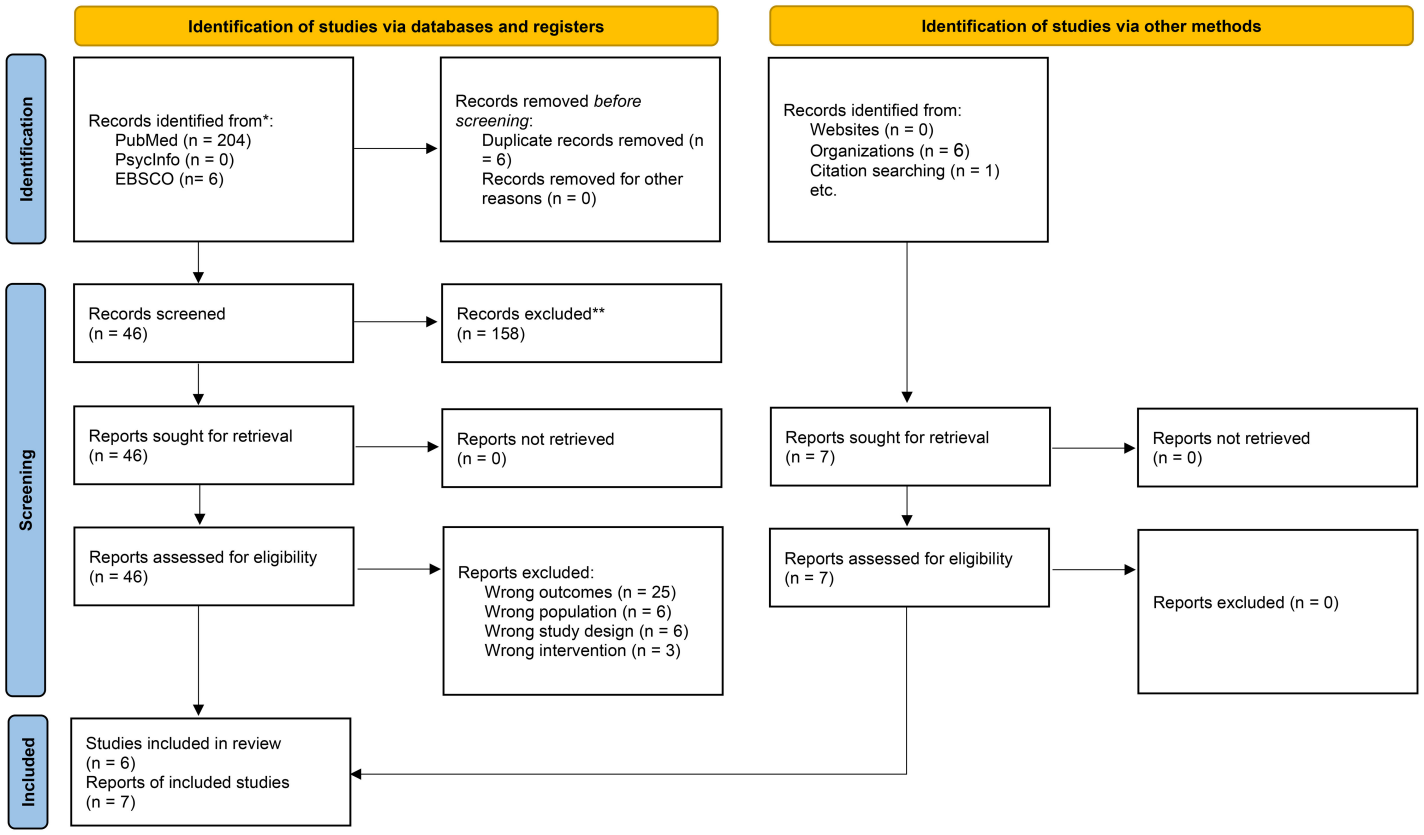
Flow diagram for searches of databases, registers and other sources.

Two independent reviewers (the first author; MMA) and (the second author; NZA) screened all potential records retrieved through the search process. Initial screening was based on titles and abstracts. Full texts were retrieved for records that met the eligibility criteria or required further evaluation. Discrepancies during screening or full-text review were resolved through discussion or consultation with a third reviewer (the last author; MMA) when needed. The selection process is illustrated using a PRISMA-style flow diagram (see **Figure 1**). We extracted data using a standardized data extraction sheet. Data extracted included: study characteristics (author and year of publication), study design, as well as sample characteristics including age, sex, and percentage receiving treatment. Further, data extracted included treatment regimen and stimulant class (i.e., MPH or AMPH based stimulants). Finally, main findings related to AMPH availability in Saudi Arabia was reviewed for all included articles.

### Data analysis and synthesis

In this narrative review, we conducted a qualitative synthesis from all included studies report AMPH-based stimulant availability for individuals with ADHD in Saudi Arabia. We conducted a qualitative narrative synthesis of the included sources, structured around three analytical domains:

1. Current prescribing and regulatory landscape for AMPH psychostimulants in Saudi Arabia.
2. Clinical practice context, including treatment guidelines and access pathways for AMPH treatment.
3. Systemic and policy-related barriers limiting availability and utilization of AMPH-based medications.

We reported our findings regarding: (i) study characteristics: authors (year), and study design; (ii) primary findings relevant to AMPH availability in Saudi Arabia; and (iii) clinical practice protocols and guidelines for individuals with ADHD in Saudi Arabia. Key findings were organized into thematic summaries with representative examples from included sources. No quantitative synthesis (meta-analysis) was performed due to the heterogeneity of study types and the nature of the data. Grey-literature sources were appraised based on their origin (e.g., government agency, academic society), internal consistency, relevance to the review question, and publication recency. Priority was given to documents issued by authorities that play key roles in ADHD diagnosis and management (e.g., Ministry of Health), or recognized scientific bodies. We recorded the issuing institution, year, and content scope for each document (**Table 1**).

**Table 1:** Participant and study characteristics of the included articles.

Author (year)	Sample size (with ADHD)	Age (years) *M* ± SD	Female sex (%)	% Receiving treatment	Study design	Treatment regimen	Stimulant class
Al-Mohsen (2020) (32)	132	4.0 ± 1.7	NR	62.1	Cross-sectional	Behavioral, pharmacological, or both	NR
Alghamdi (2022) (40)	245	21.4 ± 1.8	43.3	2.0	Cross-sectional	Pharmacological	NR
Al-Haidar (2003) (34)	105	NR	22.6	23.6	Retrospective cross-sectional	Pharmacological	MPH
Alsubaie (2024) (33)	221	NR	21.5	NR	Cross-sectional	Pharmacological	MPH and AMPH
Faraone (2021) (19)	**-**	–	–	–	Consensus statement	–	–
Chan (2023) (41)	**-**	–	–	**-**	Longitudinal trend	–	–
Bashiri (2021) (35)	**-**	–	–	**-**	Adaptation of CPGs	Behavioral, pharmacological, or both	MPH and AMPH
Ministry of Health (2019) (42)	**-**	–	–	–	CPGs	Behavioral, pharmacological, or both	MPH
Saudi ADHD Society (2020) (37)	**-**	–	–	**-**	CPGs	Behavioral, pharmacological, or both	MPH and AMPH
Saudi ADHD Society (2025) (38)	**-**	–	–	**-**	Society Work	Pharmacological	MPH and AMP
Saudi ADHD Society	**-**	–	–	**-**	Society Work	Pharmacological	MPH
Saudi ADHD Society	**-**	–	–	**-**	Society Work	Pharmacological	MPH and AMP
Council of Health Insurance (2024) (43)	**-**	–	–	**-**	Indication update	Pharmacological	MPH and AMP

MPH, Methylphenidate-based stimulants; AMPH, Amphetamine-based stimulants; CPGs, Clinical practice guidelines. NR, Not Reported; M, means; SD, Standard Deviation.

## Results

### Search results

A total of 211 records were identified of which 204 records sourced from PubMed, six records from EBSCO, and one through citation search (manual search). Additionally, six records were identified from organizations’ work. After removing six duplicate records, a total of 205 sources remained for screening. During the title and abstract screening phase, 158 records were excluded due to irrelevance to our study’s aim, leaving 46 records for the full-text screening phase. Out of the 46 articles, 25 reports were excluded for reporting wrong outcomes, six reports included wrong populations, six reports used ineligible study design, and 3 reports investigations from wrong intervention, yielding 6 reports that were included in the analysis from the databases search. In addition, one report was included by the citation manual search and six reports were included from organizations’ work. The total number of articles/reports that were included in the final analysis is 13 (19, 32–43) (**Figure 1**).

### Study characteristics

In **Table 1**, we summarize the main characteristics of the included study. Characteristics of the included studies/reports vary significantly in terms of sample size, study design, and/or treatment regimens. The studies that included participants’ data (n=4) had sample sizes varied between 105 and 245 individuals with ADHD, with all studies using a cross-sectional study design. The remaining studies consisted of consensus statements (19), longitudinal trend analyses (41), and guideline adaptations (35, 42, 43). The cross-sectional studies included children, adolescents, and adults with ADHD. Specifically, Al-Mohsen et al. (2020) (32) included young children (*M* age= 4.0 ± 1.7 years), whereas Alghamdi et al. (2022) (40) focused on older participants (*M* age= 21.4 ± 1.8 years). However, some studies, such as Al-Haidar (2003) et al. (34), included children through adolescents from early ages to 18 years, whereas Alsubaie et al. (2024) (33) included children and adults of ages less than 3 years to older than 17 years. The proportion of female participants in the included studies varied, although some studies did not report this information (sex distribution).

The percentage of participants receiving treatment also differed remarkably. Specifically, Al-Mohsen et al. (2020) (32) reported a relatively high treatment rate (62.1%), while Alghamdi et al. (2022) (40) found that only 2.0% of their sample was receiving pharmacological treatment. Al-Haidar et al. (2003) (34) indicated that 23.6% of patients were on medication, and Alsubaie et al. (2024) (33) did not provide details on medication received.

In terms of treatment regimens, many studies examined pharmacological treatments (33, 34, 36, 38–40, 43), while others investigated both pharmacological and behavioral treatments (32, 37, 42). The stimulant medications reported in the studies included MPH and AMPH. One study by Bashiri (2021) (35) and guidelines provided by the Saudi ADHD Society, acknowledged the use of both MPH and AMPH which were relatively recent. Other reports, such as the MOH guidelines, focused primarily on MPH without inclusion of AMPH stimulants.

### Main findings related to AMPH availability

In **Table 2**, we summarize the main findings related to amphetamine availability for individuals with ADHD in Saudi Arabia. Al-Mohsen et al. (2020) (32) conducted a study on Saudi mothers’ perceptions of ADHD treatment. The study revealed that concerns regarding the side effects of stimulant medications were a major factor influencing treatment adherence. Furthermore, the study found that a limited number of mothers (<26%) were aware that MPH is a medication for ADHD. In addition, that study highlighted caregiver perceptions and stigma-related concerns were main barriers in managing ADHD among Saudi mothers. Alghamdi et al. (2022) (40) investigated ADHD prevalence among university students in Saudi Arabia. The findings indicated that only 2% of diagnosed students had ever received pharmacological treatment, suggesting significant under-prescription and limited access to AMPH stimulants.

**Table 2:** Summary table of the main findings related to amphetamine availability for individuals with ADHD in Saudi Arabia.

Author (year)	Main findings
Al-Mohsen (2020) (32)	Study on Saudi mothers’ perceptions of ADHD treatment. Found that fear of medication side effects was a major barrier to adherence. Furthermore, mothers were limitedly aware that MPH is a medication for ADHD.
Alghamdi (2022) (40)	Survey on ADHD prevalence in university students. Found that only 2% of students diagnosed with ADHD had ever taken medication, indicating significant under-prescription and limited access to AMPH stimulants.
Al-Haidar (2003) (34)	Retrospective review of ADHD treatment in a psychiatric clinic. Found that only 23.6% of children diagnosed with ADHD were prescribed psychostimulants, while antidepressants were more commonly used, suggesting limited availability or prescribing of amphetamine-based stimulants.
Alsubaie (2024) (33)	Study on ADHD medication adherence in Saudi children. Found that extended-release MPH was the most commonly prescribed stimulant, while AMPH stimulants were rarely prescribed. This finding suggests potential issues with AMPH stimulants availability.
Faraone (2021) (19)	Global consensus statement on ADHD treatment. Identified access to stimulant medication as a critical factor in treatment success, highlighting that restrictions on AMPH stimulants in some countries, including Saudi Arabia led to reliance on less effective alternatives.
Chan (2023) (41)	Multinational longitudinal study on ADHD medication consumption from 2015 to 2019. Found that amphetamine-based stimulants had the highest annual increase in global consumption, with limited availability in Saudi Arabia
Bashiri (2021) (35)	Review of ADHD diagnosis and treatment in Saudi Arabia. Identified a lack of standardized protocols for prescribing stimulant medications. The study also listed AMPH stimulants as a treatment option (if available), indicating a potential gap in availability.
Ministry of Health (2019) (42)	MOH protocol highlights barriers to access for ADHD medications, including the limited availability of AMPH stimulants in Saudi Arabia. The protocol emphasizes the importance of improving access and ensuring consistent medication supply.
Saudi ADHD Society (2020) (37)	The clinical practice guideline recommends MPH and AMPH (lisdexamfetamine) as primary treatments for ADHD in Saudi Arabia but acknowledges barriers to access. Discusses the impact of limited stimulant availability on treatment outcomes.
Saudi ADHD Society (2025) (38)	This report addressed concerns about ADHD medication access in Saudi Arabia. It highlighted issues such as inconsistent supply of AMPH stimulants, regulatory restrictions, and the need for policy adjustments to improve medication availability. This report also indicated the update of lisdexamfetamine by the Saudi FDA.
Saudi ADHD Society	Updated recommendations call for improved access to ADHD medications, particularly AMPH stimulants. Highlights the need for regulatory changes to support better ADHD management.
Saudi ADHD Society	This report examined challenges in adult ADHD treatment in Saudi Arabia. Noted that limited access to AMPH stimulants restricted treatment options, which force clinicians to rely on non-stimulant medications or behavioral interventions. The study called for policy reforms to enhance access.
Council of Health Insurance (2024) (43)	The 2024 indication update adds lisdexamfetamine to the CHI formulary, recognizing the need for expanded ADHD treatment options. This step came after several calls for improving access to AMPH stimulants for this population in Saudi Arabia.

MPH, Methylphenidate-based stimulants; AMPH, Amphetamine-based stimulants; CHI, Council of Health Insurance.

Al-Haidar et al. (2003) (34) conducted a retrospective patient records review of ADHD treatment in a psychiatric clinic in Saudi Arabia. The study found that only 23.6% of children diagnosed with ADHD were prescribed psychostimulants, while antidepressants were more commonly used as an alternative. Alsubaie et al. (2024) (33) examined ADHD medication adherence of children in Saudi Arabia. The study found that extended-release MPH was the most commonly prescribed stimulant, while AMPH stimulants were rarely prescribed. Further, this study demonstrated that prescriber behavior, including concerns about medication side effects and parental hesitation may have an effect on prescribing AMPH stimulants.

Faraone et al. (2021) (19) identified access to stimulant medication as a critical factor for successful treatment outcomes. It highlighted that restrictions on AMPH stimulants in some countries, including Saudi Arabia, have led to increased reliance on non-stimulant alternatives, which may not be as effective. Bashiri et al. (2021) (35) reviewed ADHD diagnosis and treatment in Saudi Arabia and adapted the NICE guidelines related to ADHD diagnosis and management. This study identified a lack of standardized protocols for prescribing stimulant medications in Saudi Arabia. The study also listed AMPH stimulants as a treatment option (if available), indicating a potential gap in availability. Moreover, the study indicated other barriers to AMPH prescription, including clinical uncertainty and provider variability. Chan et al. (2023) (41) conducted a multinational longitudinal study on ADHD medication consumption from 2015 to 2019. The study found that AMPH -based stimulants had the highest annual increase in global consumption. However, their availability remained limited in Saudi Arabia, due to regulatory restrictions and supply inconsistencies.

The 2019 MOH Protocol for ADHD management (42) outlined several barriers to accessing ADHD medications in Saudi Arabia, including the limited availability of AMPH stimulants. The protocol emphasized the need for improved access and consistent medication supply to ensure effective ADHD management. The Saudi ADHD Society CPG (2020) (37) recommended MPH and AMPH stimulants (lisdexamfetamine) as primary treatments for ADHD in Saudi Arabia. However, the guidelines acknowledged existing barriers to accessing these medications and discussed the consequences of limited stimulant availability on treatment outcomes. The Council of Health Insurance (CHI) (2024) Indication Update (43) added lisdexamfetamine to the CHI formulary, recognizing the need for expanded ADHD treatment options. This step came after several calls for improving access to AMPH stimulants for this population in Saudi Arabia.

The Saudi ADHD Society has published three reports regarding AMPH limited availability (36, 38, 39). The first was the Saudi ADHD Society Updated Recommendations (44), which called for improved access to ADHD medications, particularly AMPH stimulants. This report highlighted the need for regulatory changes to support better ADHD management in Saudi Arabia. The second report of the Saudi ADHD Society (Adult Treatment) (36) examined challenges in adult ADHD treatment in Saudi Arabia. This report noted that limited access to AMPH stimulants restricted treatment options, which force clinicians to rely on non-stimulant medications or behavioral interventions. The report called for policy reforms to enhance access. The third report by the Saudi ADHD Society (Medication Availability FAQ) (38) addressed concerns about ADHD medication access in Saudi Arabia. It highlighted issues such as inconsistent supply of AMPH stimulants, regulatory restrictions, and the need for policy adjustments to improve medication availability. This report also indicated the update of lisdexamfetamine by the Saudi FDA.

### Clinical practice protocols/guidelines

Both the MOH (42) and the Saudi ADHD Society (37) Clinical Practice Guidelines (CPGs) acknowledge that stimulant medications, specifically MPH and AMPH, are the primary pharmacological treatments for ADHD. The MOH Protocol notes that while MPH (e.g., Ritalin) is widely available and used as the first-line treatment, AMPH stimulants such as lisdexamfetamine and dexamfetamine are not as commonly prescribed. Additionally, the protocol highlights that the off-label use of second-generation antipsychotics (e.g., Risperidone, Aripiprazole) has increased, which may be partly due to barriers in accessing AMPH stimulants. Similarly, the Saudi ADHD Society CPG acknowledges that MPH is the most commonly used stimulant in Saudi Arabia, whereas AMPH stimulants such as lisdexamfetamine and dexamfetamine are less frequently prescribed. This CPG also states that AMPH stimulants should be considered for patients who do not respond adequately to MPH stimulants. Despite their efficacy, regulatory hurdles and concerns about potential misuse and diversion have made the use of AMPH stimulants more restrictive.

Both the MOH (42) and the Saudi ADHD Society CPGs (37) emphasize the necessity of AMPH stimulants for effective ADHD management. The MOH Protocol asserts that stimulant medications remain the most effective pharmacological treatment for ADHD, particularly for individuals who exhibit moderate to severe symptoms that interfere with daily functioning. The protocol further highlights that when MPH is ineffective or poorly tolerated, AMPH should be considered as an alternative. The Saudi ADHD Society CPG reinforces the need for expanded access to AMPH stimulants (37), citing evidence from international ADHD treatment guidelines, such as NICE and the American Academy of Pediatrics (AAP), that support their efficacy. They also stress that untreated or poorly managed ADHD can lead to poor academic performance, increased risk of comorbid psychiatric disorders, and higher rates of substance misuse and criminal behavior. Thus, the Saudi ADHD Society guideline (37) strongly recommends improving access to AMPH treatments to ensure optimal patient outcomes. Additionally, the Saudi ADHD Society CPG calls for regulatory changes to facilitate the approval and distribution of AMPH stimulants, ensuring that they are available as part of the national ADHD treatment framework. A few years after this call, the Saudi FDA has approved lisdexamfetamine for patients with ADHD in public and private healthcare settings.

## Discussion

This narrative review highlights significant barriers to the availability of AMPH stimulants for individuals with ADHD in Saudi Arabia. The findings indicate that MPH is the most commonly prescribed stimulant, whereas AMPH, such as lisdexamfetamine and dexamfetamine, remain underutilized due to regulatory restrictions and supply limitations (32, 33). In addition, studies analyzing ADHD medication trends in Saudi Arabia have consistently reported low prescription rates of AMPH stimulants (41), with evidence suggesting that many patients lack access to optimal pharmacological treatment (34, 40). Furthermore, Saudi guideline for ADHD treatment, including the MOH Protocol and Saudi ADHD Society Protocol, indicated the critical role of stimulants in ADHD management but emphasized that the availability constraints continue to impact patient care (37, 42). International treatment guidelines, including NICE and AAP, recommend AMPH stimulants as effective treatments for ADHD (25, 26). Nonetheless, regulatory challenges in Saudi Arabia limit the availability of these medications, leading to increased reliance on less effective alternatives such as non-stimulant medications and off-label antipsychotic prescriptions (35). Recently, CHI’s 2024 indication update, which added lisdexamfetamine to the national formulary, represents a significant step forward in expanding treatment options (43). In fact, there is a dire need to expand the categories of AMPH stimulants, such as mixed amphetamine salts (Adderall), to ensure equitable access to evidence-based treatments for ADHD in Saudi Arabia.

The availability and regulation of AMPH stimulants for ADHD vary widely across countries, with notable differences between Saudi Arabia, the United States (US), the United Kingdom (UK), and other Gulf Cooperation Council countries. In the US, AMPH stimulants such as mixed amphetamine salts (Adderall) and lisdexamfetamine (Vyvanse) are widely prescribed as treatments for ADHD, following the AAP guidelines (25). In the UK, AMPH stimulants such as dexamfetamine and lisdexamfetamine are also included in NICE guidelines as recommended treatments for ADHD, particularly for patients who do not respond to MPH (26). However, both the US and the UK implemented stringent prescription and monitoring protocols to mitigate risks of misuse (25, 45). In Gulf countries such as the United Arab Emirates (UAE) and Kuwait, the availability of AMPH stimulants is also restricted (41). While the SFDA recently approved lisdexamfetamine for individuals with ADHD, challenges remain in ensuring widespread availability and physician prescribing confidence (38). Standard healthcare from evidence-based CPGs suggests that balancing regulatory control with improved access through specialist prescribing programs and prescription monitoring systems could improve ADHD management in Saudi Arabia.

The restricted availability of AMPH stimulants for ADHD in Saudi Arabia has significant impact on treatment outcomes. One of the primary concerns is the delay in initiation of effective pharmacological interventions. Research has shown that early intervention with psychostimulant medications, including AMPH, leads to enhanced academic performance, improved social functioning, and reduced risk of comorbid psychiatric disorders (19). However, due to the limited availability of these medications, many patients in Saudi Arabia may experience prolonged untreated ADHD symptoms, potential risks exist pertaining to academic underachievement, occupational difficulties, and social functioning.

The lack of AMPH has also resulted in increased reliance on off-label or non-stimulant medications. Patients who do not respond well to MPH often receive non-stimulant alternatives such as atomoxetine or even second-generation antipsychotics (40, 42). While non-stimulants can be effective in certain cases, they have a lower effect size compared to stimulant medications and may not provide sufficient symptom control for all individuals with ADHD (46). This situation forces clinicians to prescribe medications that may not align with the gold standard of ADHD treatment recommended by national and international CPGs.

Beyond regulatory limitations, multiple psychosocial and cultural factors contribute to reduced AMPH prescribing in Saudi Arabia. Researchers highlighted other barriers to prescribing AMPH, including physician reluctance, medicolegal risk, lack of stimulant prescribing experience, and concerns over perceived overdiagnosis (33) (35). Public stigma toward ADHD and psychostimulants was also described as a major barrier to family acceptance of medication (32). Such contextual barriers may explain the variability in prescribing rates, suggesting the need for both educational outreach programs.

Recent international studies have raised concerns that low stimulant consumption relative to diagnosed ADHD prevalence may signal under prescription or inappropriate treatment practices, particularly when low-psychostimulant consumption relative to non-stimulants is present. For example, a national-wide study from Slovenia (47) reported a decline of stimulant use in children and adolescents despite the increased ADHD diagnosis. This example highlights the need to analyze trends of ADHD treatment in Saudi Arabia, considering the ratio of stimulant to non-stimulant prescription.

Despite the growing body of literature on ADHD treatment, significant research gaps remain, particularly concerning the availability and efficacy of AMPH medications in Saudi Arabia. Addressing these gaps in future studies is crucial for optimizing ADHD management and ensuring equitable access to effective pharmacological treatments. One critical area of future research is conducting epidemiological studies to assess ADHD treatment gaps in Saudi Arabia. Although national data on ADHD prevalence exists for children, adolescents, and adults, there is limited information regarding the proportion of patients who receive optimal pharmacological treatment. Studies have shown that stimulant medications, particularly AMPH, are under-prescribed in Saudi Arabia compared to other countries (34, 40). Future research should explore the factors contributing to these disparities, such as regulatory restrictions, physician prescribing behaviors, and public perceptions of ADHD medications. Understanding these barriers through large-scale epidemiological surveys will provide valuable insights for policymakers to implement targeted interventions aimed at improving medication accessibility.

Investigating healthcare provider perspectives on psychostimulant prescriptions is essential for understanding prescribing patterns and potential hesitancies in using amphetamines for ADHD treatment. Previous research has indicated that concerns over misuse, addiction potential, and regulatory constraints influence prescribing behaviors among clinicians (35). Qualitative studies involving psychiatrists, pediatricians, and primary care physicians could help elucidate the factors that shape prescribing preferences and identify strategies to enhance physician confidence in managing ADHD with a broader range of stimulant options. Additionally, studies examining the impact of continuing medical education programs on psychostimulant prescription practices may provide insights into how physician training can improve evidence-based prescribing behaviors.

### Study limitations

Several limitations should be acknowledged in this narrative review. First, potential biases may have been introduced during the identification, selection, and interpretation of the included literature. These biases could stem from the selective inclusion of studies, the inclusion of grey literature, or subjective judgment in evaluating the findings. As typical in narrative reviews, the interpretation of evidence and selection of themes may reflect author judgment rather than objective appraisal. Furthermore, the inclusion of grey literature and policy documents, although valuable, may have introduced variability in the quality and reliability of the information. Finally, the narrative approach allowed inclusion of policy documents and introduced subjectivity inherent in qualitative synthesis of evidence. Additionally, due to the nature of the included grey literature, reviewers were not blinded to document origin, which may introduce bias despite our use of independent screening and data verification. Therefore, the findings should be interpreted with caution.

## Conclusion

This narrative review highlights the significant challenges surrounding the availability of AMPH stimulants for ADHD in Saudi Arabia. While MPH remains the most commonly prescribed stimulant, access to AMPH, including lisdexamfetamine and mixed amphetamine salts, remains limited in Saudi Arabia due to several factors, such as regulatory constraints and concerns about misuse. The limited access to these medications has critical implications for ADHD management, including delayed treatment and reliance on less effective treatment alternatives. Preliminary evidence supports the need for policy attention to AMPH availability. These policies should focus on reassessing psychostimulant medication efficacy and implementing robust prescription monitoring systems. Future research should focus on addressing treatment gaps through epidemiological studies and clinical trials to compare different psychostimulant classes.

## Data Availability

The original contributions presented in the study are included in the article/**Supplementary Material**. Further inquiries can be directed to the corresponding author.

## Supplementary material

Supplementary Table 1 Search terms and database used in this narrative review.
