# Supplementary material for: Challenges in amphetamine medication availability for individuals with ADHD: a narrative review of the current state of evidence
Source: Front Psychiatry. 2025 Jul 23;16:1624590. doi: 10.3389/fpsyt.2025.1624590 (PMC12326170; doi:10.3389/fpsyt.2025.1624590)
Supplement: Supplementary Table 1 — Search terms and database used in this narrative review. [file Table1.pdf]

Supplement 1. Search terms and database used in this narrative review.

Database	Search Terms	Number of Retrieved Articles
PubMed	((("amphetamine s"[All Fields] OR "amphetamines"[MeSH Terms] OR "amphetamines"[All Fields] OR "amphetaminic"[All Fields] OR "dextroamphetamine"[MeSH Terms] OR "dextroamphetamine"[All Fields] OR "amphetamine"[All Fields] OR "amphetamine"[MeSH Terms] OR ("dextroamphetamine"[MeSH Terms] OR "dextroamphetamine"[All Fields] OR "dexamphetamine"[All Fields]) OR ("psychostimulant"[All Fields] OR "psychostimulants"[All Fields] OR "psychostimulating"[All Fields] OR "psychostimulation"[All Fields] OR "psychostimulator"[All Fields] OR "psychostimulators"[All Fields]) OR ("central nervous system stimulants"[Pharmacological Action] OR "central nervous system stimulants"[MeSH Terms] OR ("central"[All Fields] AND "nervous"[All Fields] AND "system"[All Fields] AND "stimulants"[All Fields]) OR "central nervous system stimulants"[All Fields] OR "stimulants"[All Fields] OR "stimulant"[All Fields])) AND ("attention deficit disorder with hyperactivity"[MeSH Terms] OR ("attention"[All Fields] AND "deficit"[All Fields] AND "disorder"[All Fields] AND "hyperactivity"[All Fields]) OR "attention deficit disorder with hyperactivity"[All Fields] OR ("attention"[All Fields] AND "deficit"[All Fields] AND "hyperactivity"[All Fields] AND "disorder"[All Fields]) OR "attention deficit hyperactivity disorder"[All Fields])) OR ("attention deficit disorder with hyperactivity"[MeSH Terms] OR ("attention"[All Fields] AND "deficit"[All Fields] AND "disorder"[All Fields] AND "hyperactivity"[All Fields]) OR "attention deficit disorder with hyperactivity"[All Fields] OR "adhd"[All Fields])) AND ("saudi arabia"[MeSH Terms] OR ("saudi"[All Fields] AND "arabia"[All Fields]) OR "saudi arabia"[All Fields])	204
PsychInfo	(Any Field: amphetamine OR Any Field: psychostimulant OR Any Field: dextroamphetamine) AND (Any Field: ADHD OR Any Field: attention deficit hyperactivity disorder) AND (Any Field: saudi arabia)	0
EBSCO	(ADHD OR attention deficit hyperactivity disorder) AND (psychostimulants OR amphetamine OR dexamphetamine) AND Saudi Arabia	6
